# Monitoring Activity and Gait in Children (MAGIC) using digital health technologies

**DOI:** 10.1038/s41390-024-03147-x

**Published:** 2024-03-21

**Authors:** Junrui Di, Pirinka Georgiev Tuttle, Lukas Adamowicz, Wenyi Lin, Hao Zhang, Dimitrios Psaltos, Jessica Selig, Jiawei Bai, F. Isik Karahanoglu, Paul Sheriff, Vijitha Seelam, Bunmi Williams, Sana Ghafoor, Charmaine Demanuele, Mar Santamaria, Xuemei Cai

**Affiliations:** 1 Pfizer, Inc., Cambridge, MA USA; 2 Tufts Medical Center, Boston, MA USA

## Abstract

**Background:**

Digital health technologies (DHTs) can collect gait and physical activity in adults, but limited studies have validated these in children. This study compared gait and physical activity metrics collected using DHTs to those collected by reference comparators during in-clinic sessions, to collect a normative accelerometry dataset, and to evaluate participants’ comfort and their compliance in wearing the DHTs at-home.

**Methods:**

The MAGIC (Monitoring Activity and Gait in Children) study was an analytical validation study which enrolled 40, generally healthy participants aged 3–17 years. Gait and physical activity were collected using DHTs in a clinical setting and continuously at-home.

**Results:**

Overall good to excellent agreement was observed between gait metrics extracted with a gait algorithm from a lumbar-worn DHT compared to ground truth reference systems. Majority of participants either “agreed” or “strongly agreed” that wrist and lumbar DHTs were comfortable to wear at home, respectively, with 86% (wrist-worn DHT) and 68% (lumbar-worn DHT) wear-time compliance. Significant differences across age groups were observed in multiple gait and activity metrics obtained at home.

**Conclusions:**

Our findings suggest that gait and physical activity data can be collected from DHTs in pediatric populations with high reliability and wear compliance, in-clinic and in home environments.

**Trial registration:**

ClinicalTrials.gov: NCT04823650

**Impact:**

- Digital health technologies (DHTs) have been used to collect gait and physical activity in adult populations, but limited studies have validated these metrics in children.
- The MAGIC study comprehensively validates the performance and feasibility of DHT-measured gait and physical activity in the pediatric population.
- Our findings suggest that reliable gait and physical activity data can be collected from DHTs in pediatric populations, with both high accuracy and wear compliance both in-clinic and in home environments.
- The identified across-age-group differences in gait and activity measurements highlighted their potential clinical value.

## Introduction

Gait assessments provide key insights for clinicians to assist in understanding an individual’s mobility and their overall health. In recent years, instrumented gait analyses using digital health technologies (DHTs) have been shown to be able to collect objective data for diagnosis and/or monitoring across a wide range of diseases and populations.^1^ These assessments also provide insights into the emergence and progression of health conditions. Gait has been studied in children across psychiatric, neurological, and motor diseases.^2–10^ The European Medicines Agency (EMA) recently accepted stride velocity 95th percentile as a primary endpoint in Duchenne Muscular Dystrophy (DMD) clinical trials.^11^

In addition to gait, physical activity is another key aspect to monitor in developing children. The benefit of physical activity is well defined: obesity prevention,^12,13^ reduction of cardiovascular risk factors,^14,15^ normal growth and development,^16,17^ depression prevention,^18^ reduction in risk of chronic diseases, health-related quality of life.^19^ One recent neuroimaging study found an association between physical activity in late childhood (ages 10–14 years) and increased volumetric changes in brain structures such as the amygdala.^20^

Physical activity and gait can be measured with DHTs, specifically wearable accelerometers, which provide continuous, passive data collection and potentially a broader representation of how individuals function over time.^21^ Studies involving DHTs in pediatric populations have found associations between age and gait metrics,^22–27^ while other variables such as gender and sex have not shown a strong correlation.^27,28^

Using DHTs to measure gait and physical activity in clinical trials requires a body of evidence for acceptance^29^ and validation.^30^ in the population of interest. Despite existing clinical research, there remains a lack of standardized measures and further validation of DHTs in developing children.^27,31^ The validation of such DHTs could serve as a reference for the development of technologies designed for children with specific conditions.^7,32–34^ While there is interest in the field of expanding the use of DHTs in children, only a few studies comprehensively assessed the feasibility of implementation.^35^ Of those, the ActiGraph devices have been investigated in several studies, involving children ages 2–18.^36–41^

In this paper, we share the results from the Monitoring Activity and Gait in Children (MAGIC) study, a non-randomized low-interventional study in generally healthy pediatric participants between the ages of 3–17 years, as a comprehensive evaluation of DHT-measured gait and physical activity in pediatric participants. The main objective of the study was to validate the gait metrics derived from the in-house built SciKit Digital Health (SKDH) Gait Module^42^ using data collected from lumbar-worn DHTs (ActiGraph) against ground truth gait measurements (GAITRite^®^ walkway), in a clinical setting. In addition, wear compliance and comfortability data were analyzed to evaluate the overall usability of DHTs in children. The difference between gait and physical activity measured in-clinic and at home were also compared. Lastly, differences in gait and physical activity across the three different age groups were evaluated.

## Methods

### Protocol and study design

The MAGIC study was a non-randomized, low-interventional analytical validation study to evaluate gait and physical activity measured by DHTs, specifically wearable accelerometers. A total of 40 healthy ambulatory participants aged between 3 and 17 years were enrolled in the study, to ensure enough statistical power to estimate the agreement between DHT-measured gait and reference. The participants were divided into three independent age groups as follows: 3–5 years (*n* = 13), 6–11 years (*n* = 14), and 12–17 years (*n* = 13). Informed consent was obtained from the parents/legally acceptable representatives of all participants. Participants provided their assent consistent with their age (verbally for children aged 3–5 years, written for children aged 6–17 years). The study was conducted at the Pfizer Innovation Research Lab (PfIRe Lab) in Cambridge, Massachusetts from 2021 to 2022. Participants were recruited through paper and electronic flyers, internet postings, and advertisements at community centers. Ethics approval of the protocol was obtained from Advarra Institutional Review Board (protocol number: Pro00047861). The study was registered at ClinicalTrials.gov under the identifier NCT04823650.

The MAGIC study included two components, in-clinic with multiple controlled walking tasks and simulated activities, and ~2 weeks of continuous at-home monitoring. Only study procedures relevant to this work were presented here. During the in-clinic portion, participants were asked to perform staged walking tasks on an instrumented walkway (GAITRite^®^, CIR systems Inc., Franklin, New Jersey) at natural, fast, and slow speeds, each repeated three times. The natural walk was described as regular, normal speed. This was also used to explain to the children later to increase their speed for the fast walk and decrease for the slow walk. Children were asked not to jump or skip while walking and the site staff demonstrated the differences. Gamification was implemented for younger children with the incorporation of games such as “the floor is lava” to encourage walking only on the mat, and “red light green light” along with toys and wall decals which were used to denote where to start and stop walking the full length of the mat. For all tasks, the main DHTs used were two ActiGraph Centrepoint Insight Watches (CPIW) (ActiGraph; Pensacola, Florida; dimensions: 5 × 3.43 × 1.04 cm; weight: 19 g), one worn on the lower back (lumbar region), and the other on the non-dominant wrist; both were configured to collect data at a sample rate of 64 Hz. Participants were also asked to respond to a comfort and wearability questionnaire for the devices used at the end of the clinic visit. For children 3–11 years old, the parents answered the questions, and children 12 years and older answered the questions themselves. The comfort and wearability questionnaire contained ten questions such as whether the specific DHT was overall comfortable, the DHT was easy to put on or take off, and the participants’ willingness to wear the DHTs continuously etc. For each question, participants chose from “strongly disagree”, “disagree”, “neutral”, “agree”, and “strongly agree”. Details of the questionnaire can be found in supplementary material (Supplementary Tables 1, 2).

During the at-home portion, participants wore two ActiGraph CPIWs, one on the non-dominant wrist and one on the lumbar region, for ~2 weeks. In-person verbal instructions were provided to each participant prior to leaving the clinic. Specifically, they were instructed to wear the wrist DHTs continuously, and to put on the lumbar DHTs when they woke up in the morning/got up from bed and to remove it when they went to sleep at night or napped during the day. At the end of the at-home period, a follow-up phone call was conducted for the participant to respond to a comfort and wearability questionnaire (same as above) for the DHTs. The DHTs were mailed back to site by participants/parents/caregivers at the end of the study.

### Gait and physical activity algorithms

The gait algorithm module from SKDH Python package^42^ (version 0.11.1) was used to extract gait metrics. First, wavelet transforms were used to detect initial and final contact events for each foot from the vertical acceleration signal,^43^ followed by the computation of all temporal metrics (e.g., stride time, double support, etc.) Then, an inverted pendulum model of gait was used to extract bilateral spatial gait characteristics from the acceleration data.^43–46^ For spatial metrics such as stride length and gait speed, the inverted pendulum model was used with participants’ heights as an input. For free-living accelerometry data, i.e., when the time periods of gait were unknown, a gradient boosted tree classifier was applied first to detect the gait bouts before the derivation of gait characteristics.

The physical activity metrics were derived using the Crouter algorithm implemented in the ActiGraph CentrePoint Version 3 API (https://github.com/actigraph/CentrePoint3APIDocumentation).^47,48^ The Crouter algorithm uses regression analyses to develop prediction equations for energy expenditure (metabolic equivalent of task) and cut points for computing activity metrics such as time spent in sedentary behaviors, light, moderate, and vigorous physical activity.

### Statistical analysis

For the validation analyses, we focused on key gait metrics including stride duration, stride length, cadence, and gait speed, as well as key physical activity metrics including, moderate to vigorous physical activity (MVPA), sedentary behavior, and total vector magnitude.

For in-clinic walking tasks at different speeds, gait metrics derived from SKDH-gait using lumbar-worn ActiGraph data were compared to GAITRite®, as reference, using intraclass correlation coefficients (ICC) and Pearson correlation coefficients. Specifically, ICC_2,1_ (two-way random effects, absolute agreement, with respect to single measurement) were used according to the following benchmarks: ICC ≤ 0.4 indicates “poor”, 0.4–0.59 “moderate”, 0.6–0.74 “good”, and 0.75–1 “excellent’ agreement”.^49^

Wearability and comfort questionnaires from wrist and lumbar-worn DHTs were summarized for in-clinic and at-home portions, separately. Total scores were calculated for both the wrist and lumbar-worn DHTs for in-clinic and at-home portions to represent the overall comfort and wearability score. The total score ranged between 0 to 40 where 0 means worst comfortability while 40 means the best comfortability. Details of the scoring methods can be found in Supplementary Tables 1, 2. A higher score indicates higher overall comfortability and user acceptability. Scores from wrist- and lumbar-DHTs were compared using Paired Wilcoxon Signed Rank to determine the preferred location for device placement. Furthermore, wear time and compliance for both the wrist and lumbar-worn DHTs were summarized. Wear time was derived using the Choi algorithm.^50^ During the at-home portion, compliance for lumbar-worn DHTs was defined as wearing for at least 10 h a day. Compliance for wrist-worn DHTs was defined as wearing for at least 18 h a day. Percent of compliance was calculated as compliant days divided by the total number days during the at-home portion.

For the gait data collected at-home using lumbar-worn DHTs, only days with at least 10 h of wear time were included in the analyses. To account for outliers, which are upper and lower extremes in bout length, bouts that lasted <10 s or >3000 s were excluded from the analysis.^51^ Moreover, only bouts with at least four detected gait cycles were included in the analyses to ensure robust gait metrics estimation.^51^ For each participant, the median per walking bout was taken for each gait metric, followed by the mean across all walking bouts within each day of measurements. In addition, 95^th^ percentile gait speed, which has been shown recently to have the potential to be used as a primary endpoint in clinical trials,^52^ was also computed across all walking bouts within each day of measurements. For the physical activity data collected using wrist-worn DHTs, only days with at least 18 h of wear time were included in the analyses. For the following analyses, participants with four or more valid compliant days were included. No missing imputation was considered. Mixed model with repeated measurements (MMRM) was used to evaluate the difference between in-clinic measurements and at-home measurements of gait, where each gait measurement was fitted as outcome, and in-clinic vs. at-home as the main factor, while adjusting for age groups. For such comparisons, the gait estimated from the natural walking task were used to represent the in-clinic measurements. Analysis of variance (ANOVA) was used to evaluate the effects of age groups on gait and physical activity data collected at-home with pair-wise comparison included between age groups.

## Results

### Population and recruitment

82 individuals were contacted to participate in the study and 40 were enrolled. All participants completed the study with no discontinuation, and with 13 participants in the 3–5 years age group, 14 in the 6–11 years age group, and 13 in the 12–17 years age group. Details of the demographic characteristics can be found in Table 1. There were 22 females and 18 males, with a BMI of 18.86 ± 3.79 and age of 9.38 ± 4.48 years. The 3–5 years age group took the longest to recruit (232 days), and the shortest to recruit was the 12–17 years group (77 days).

**Table 1:** Demographic characteristics.

	3–5 years old (*N* = 13)	6–11 years old (*N* = 14)	12–17 years old (*N* = 13)	Total (*N* = 40)
Age (Years), *n* (%)				
*n*	13	14	13	40
Mean (SD)	4.38 (0.65)	9.00 (1.80)	14.77 (1.59)	9.38 (4.48)
Median (range)	4.00 (3, 5)	9.50 (6, 11)	15.00 (12, 17)	9.50 (3, 17)
(Q1, Q3)	(4.00, 5.00)	(7.00, 11.00)	(14.00, 16.00)	(5.00, 14.00)
Sex, *n* (%)				
Male	6 (46.2)	8 (57.1)	4 (30.8)	18 (45.0)
Female	7 (53.8)	6 (42.9)	9 (69.2)	22 (55.0)
BMI (kg/m^2)				
*n*	13	14	13	40
Mean (SD)	16.53 (1.94)	18.25 (3.81)	21.86 (3.34)	18.86 (3.79)
Median (range)	15.63 (15, 21)	16.97 (14, 26)	20.67 (17, 28)	17.99 (14, 28)
(Q1, Q3)	(15.15, 17.16)	(14.94, 22.23)	(19.67, 25.09)	(15.38, 21.50)
Race, *n* (%)				
White	8 (61.5)	10 (71.4)	8 (61.5)	26 (65.0)
Black or African American	1 (7.7)	0	0	1 (2.5)
Asian	3 (23.1)	3 (21.4)	5 (38.5)	11 (27.5)
Multiracial	1 (7.7)	1 (7.1)	0	2 (5.0)
Ethnicity, *n* (%)				
Hispanic or Latino	2 (15.4)	1 (7.1)	2 (15.4)	5 (12.5)
Not Hispanic or Latino	11 (84.6)	11 (78.6)	11 (84.6)	33 (82.5)
Not Reported	0	2 (14.3)	0	2 (5.0)

### Gait measurements validation

**Fig. 1:**
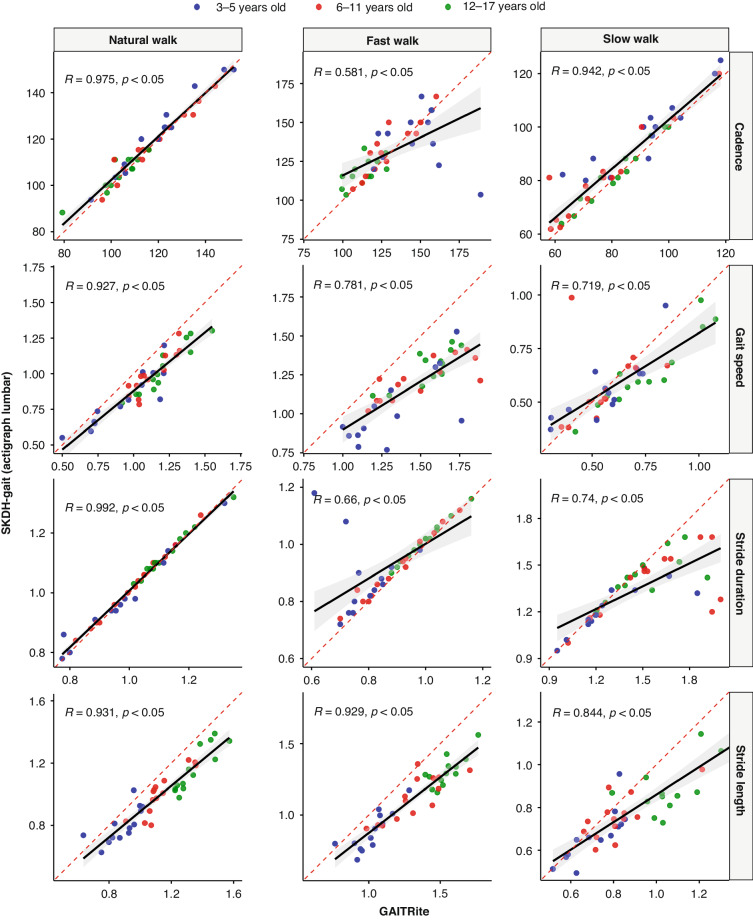
Scatter plots to show the correlation between gait metrics generated by SKDH-gait from lumbar-worn ActiGraph and GAITRite^®^ during the instrumented walking tasks at multiple speeds. R is the Pearson correlation coefficient, and *p* is the corresponding *p* value.

ICCs for in-clinic gait validation analyses for instrumented walking tasks are shown in Table 2. The four key gait metrics, i.e., cadence (steps/minute), gait speed (meters/sec), stride duration (sec), and stride length (meters), showed good to excellent agreement at both natural and slow walks (ICC ranged from 0.638 to 0.99), with the natural walk always having the best agreement between SKDH-gait and the GAITRite^®^. The agreements for all four metrics dropped for fast walk, with cadence and gait speed having only moderate agreement (0.578 and 0.432, respectively) and stride duration and stride length having good agreement (0.623 and 0.697 respectively). As shown in Fig. 1, for all four metrics across all different walking speeds, gait metrics generated by SKDH-gait showed significant linear correlation with GAITRite^®^. Consistent with the ICC estimated agreement, the natural and slow walks showed strong correlations (coefficient ranges from 0.719 to 0.992).

**Table 2:** ICC (together with upper and lower bounds) for in-clinic walking tasks.

	Natural Walk	Fast Walk	Slow Walk
Cadence	0.971 (0.94, 0.99)	0.578 (0.33, 0.75)	0.914 (0.60, 0.97)
Gait Speed	0.748 (−0.14, 0.95)	0.432 (−0.13, 0.82)	0.704 (0.50, 0.83)
Stride Duration	0.99 (0.98, 1.00)	0.623 (0.35, 0.79)	0.638 (0.29, 0.81)
Stride Length	0.773 (−0.16, 0.95)	0.697 (−0.12, 0.93)	0.721 (0.08, 0.90)

### Comfort and wearability

From the in-clinic questionnaire, 85% of the participants either “agree” or “strongly agree” that the wrist and lumbar DHTs were comfortable to wear. There was no statistically significant difference between the total score of wrist and lumbar DHTs during the in-clinic portion. The at-home questionnaire revealed that 95% of the participants either “agree” or “strongly agree” that wrist DHTs were comfortable to wear, while only 72.5% of the participants either “agree” or “strongly agree” that the lumbar DHTs were comfortable to wear at home. 97.5% and 82.5% of the participants either “agree” or “strongly agree” that they were willing to wear the wrist and lumbar accelerometers for more than 7 days, respectively. The overall score for the wrist-worn DHTs was significantly higher than the lumbar DHTs (the mean difference across participants was 2.73), indicating participants’ preference for the wrist DHTs during the at-home period. Responses from each of the in-clinic and at-home comfort and wearability questionnaires can be found in the Supplementary Material Tables 1 and 2.

Three participants developed mild-skin reactions or rash from the DHTs; all reactions resolved during the study.

### Compliance

Participants wore the wrist DHTs for 21.62 (±2.18) hours per day and wore the lumbar DHTs for 11.36 (±5.04) hours when they were awake. Based on the prespecified wearing compliance criteria, compliance was at 86.01% for the wrist DHTs and 67.65% for the lumbar DHTs at-home. Of note, the 6–11 and 12–17 age groups had 79.35% and 81.23% compliance for the lumbar DHTs, which was much higher than the 3–5 age group (41.47%).

### Difference in gait between in-clinic and at-home measurements

Mean differences between in-clinic and at-home measurements of gait estimated from MMRMs are shown in Table 3 (the difference was calculated as in-clinic measurement minus at-home measurement). We observed statistically significant differences between in-clinic and at-home measurements. In particular, cadence was significantly higher, stride length longer and gait speed higher in-clinic compared to at-home. Meanwhile the stride duration was significantly longer and the 95th percentile of gait speed significantly larger at home when compared to the in-clinic during the natural walking tasks.

**Table 3:** Mean difference between in-clinic and at-home gait measurements estimated by MMRM.

	Mean Difference and 95% CI	*P* value
Cadence	2.63 (0.97, 4.29)	0.002
Stride Duration	−0.032 (−0.047, −0.016)	<0.001
Stride Length	0.049 (0.030, 0.067)	<0.001
Gait Speed	0.071 (0.045, 0.096)	<0.001
Gait Speed 95th Percentile	−0.20 (−0.24, −0.17)	<0.001

### Age effects on gait and physical activity

**Fig. 2:**
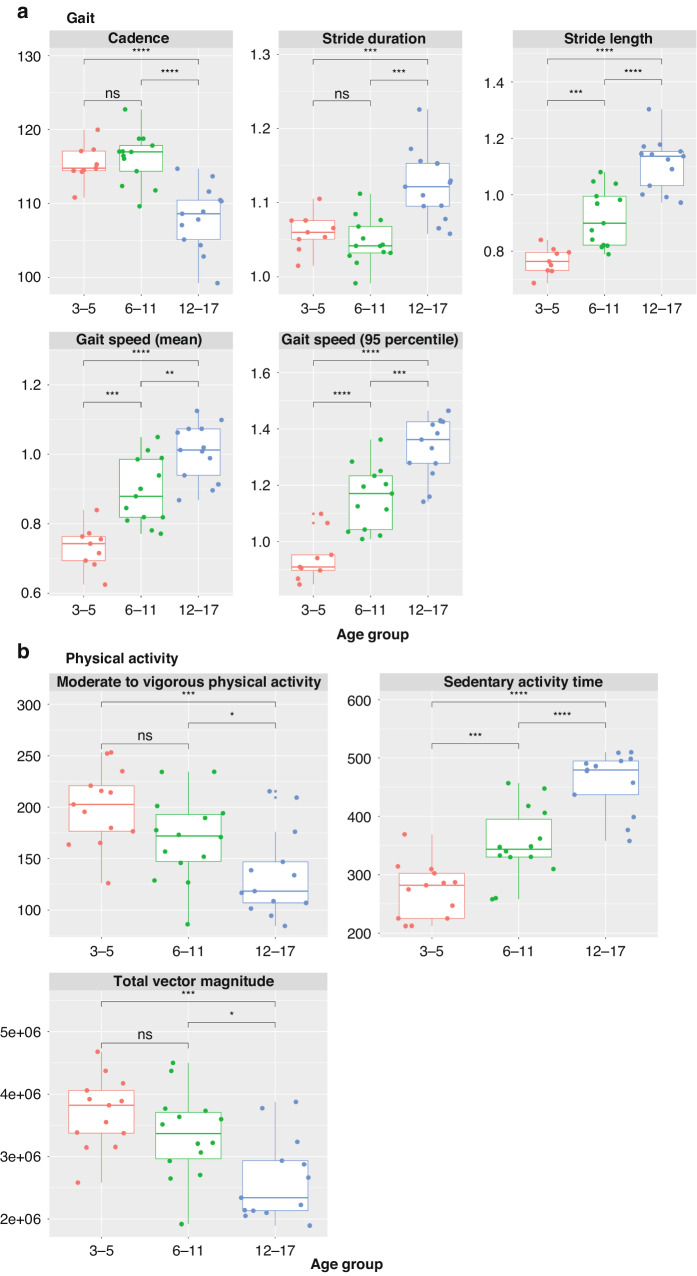
Box plots for gait and physical activity measured using the ActiGraph CPIW at-home by age groups. **a** gait and **b** physical activity. ns not significant. *p ≤ 0.05, **p ≤ 0.01, ***p ≤ 0.001, ****p ≤ 0.0001.

For measurements of gait during at-home monitoring, stride length, mean gait speed, and 95th percentile gait speed increased with age as shown in panel (a) of Fig. 2. The oldest age group (12–17) had reduced cadence, and increased stride duration compared to the younger age groups (3–5, and 6–11).

As shown in panel (b) in Fig. 2), there was a trend of decreased physical activity (measured by MVPA and total vector magnitude) and increased sedentary behavior with age. Specifically, the differences in sedentary behavior across all age groups were significant. For MVPA and total vector magnitude, with a decreasing trend, there was no statistically significant difference between 3–5 and 6–11 age groups.

## Discussion

To the best of our knowledge, the MAGIC study was one of the first pediatric studies that consisted of thorough validation of a wearable sensor-based gait characterization algorithm, i.e., SKDH-gait, for multiple in-clinic tasks, evaluation of acceptability and at-home wear compliance of DHTs, and exploration of gait and physical activity data collected in free-living environments. It may serve as a reference for future use of wrist- and lumbar-worn DHTs in pediatric clinical studies.

Excellent agreements and strong correlations were observed between SKDH-gait and GAITRite^®^ for natural and slow walks, which demonstrated good performance in SKDH-gait’s capability to characterize gait related features during these two speeds. For fast walks, the agreements between SKDH-gait and GAITRite^®^ were not as high compared to natural and slow walks. Particularly for cadence and stride duration, it was observed that the algorithm cannot provide reliable estimation for some participant-tasks, likely due to static physiological thresholds that attempt to determine unnatural or impossible steps. These thresholds were kept consistent across walking tasks to maintain generalizability, but this may adversely impact fast and slow walks. Future work may explore relaxing or making these thresholds more dynamic. For gait speed and stride length, the lower agreement is primarily due to systematic biases. Characterizing fast walk is known to be technically challenging. For example, a previous study observed higher measurement errors for fast walking in children when using an ankle sensor compared to GAITRite^®^.^53^ However, there are still significant linear correlations between SKDH-gait and GAITRite^®^ for the fast walk.

The study also showed excellent compliance in terms of wear-time by pediatric participants for wrist-worn DHTs across age groups. The compliance for the youngest cohort (3–5 years old) for lumbar-worn DHTs was particularly low. However, this may be partially due to the longer and more frequent sleep periods, since participants were instructed to remove the DHTs before sleep periods. Most participants reported that they would be willing to wear the DHTs for longer than 7 days, indicating that it is feasible to continuously collect DHT data in pediatric populations. However, other research has observed novelty effects where at first, participants were excited to wear the DHTs frequently, but lost interest after 2–4 weeks, which resulted in a decrease in overall wear-time.^54^ This indicates that further studies should investigate the long-term acceptability and feasibility of wearable DHTs in pediatric populations. In addition to willingness from children to participate in research, parental acceptance is critical for the success of pediatric clinical trials, beginning with the initial study design,^55^ even in digital health research.^56^ The physical size of the DHT and whether it is age appropriate should be considered before implementation in clinical care and trials.^57^

It should also be acknowledged that traditional outcome assessments are not always appropriate for use in younger children in interventional trials. Examples such as the 6-min walk test are not advised in children younger than 5 years old due to lack of concentration for the period required and varied results,^58–60^ as well as manual muscle testing, which cannot be reliably assessed in children younger than 5 years old who are not able to cooperate or understand.^61^ There remains an existing need for endpoints in pediatrics that are clinically meaningful, responsive to treatment or disease progression, reproducible, and reliable.^62^ Published studies in healthy children as young as 2 years old, have found value in physical activity measures obtained from wearable DHTs as potential endpoints for pediatric use, with the opportunity to use these technologies for remote monitoring.^63,64^ The results of this study pave the path for future work to validate these endpoints in younger patient populations. To date, attitudes on the acceptability and feasibility of wearable DHTs among pediatric populations and their caregivers have been positive.^7,65^ However, improvements to DHTs such as battery life.^7^ and waterproofness,^34^ and development of algorithms tailored to children with certain conditions are needed.^33^ Moreover, patient-centricity is key for trial-based drug development and must be considered. The use of DHTs such as wearable accelerometry provides opportunities for trial sponsors to consider decentralized clinical trials which can reduce patients’ burden and increase patients’ diversity.^66^

Age is a key risk factor for a wide variety of diseases and health conditions.^67^ The MAGIC study intentionally enrolled the 3 different age groups, i.e., 3–5, 6–11, 12–17, to explore the difference in gait and physical activity between age groups of actively developing children. By exploring data collected from the free-living environment, significant between-age-group differences were observed for gait and physical activity metrics. Particularly, the older group (12–17) showed statistically significant differences in characteristics of gait and physical activity as compared to the two younger age groups, which potentially demonstrated crucial developmental changes. No statistically significant differences between 3–5 and 6–11 years of age for stride duration and cadence were observed. One hypothesis is that the youngest population takes shorter strides, and their fast steps could not be captured by temporal gait measurements. Therefore, measurements like gait speed can potentially be used as a more meaningful clinical endpoint in this population. Overall, decreased physical activity and increased sedentary behaviors were observed with age. This is consistent with previous findings from a large national cohort study which showed that from childhood to adolescence, physical activity is sharply lower until age 19.^68^ This study corroborates evidence from previous research that childhood and adolescence represent a high-risk time period for physical inactivity,^69,70^ which is associated with multiple chronic diseases and comorbidities.

There are several limitations to this work. The gait validation tasks were performed in-clinic in a supervised environment. There are still challenges in acquiring and interpreting accelerometry-derived gait measurements in a free-living environment, as there will be other intercurrent events such as hospitalization that may affect gait and physical activity measurements. The validation was conducted only in healthy children and youth. This type of normative dataset can be leveraged for understanding differences in pediatric populations with specific conditions. Further validation studies should focus on generalizing the algorithms to pathological pediatric cohorts with gait and mobility disorders. We have also collected gait and physical activity measurements in children with achondroplasia enrolled in an observational trial (NCT03794609) and those with DMD undergoing treatment (NCT03362502), where healthy children in MAGIC can be used for comparison analyses and to establish construct validity for validation of these measures. The age effects observed in this study were based on the cross-sectional design of the MAGIC study. Longitudinal studies are needed to confirm these findings. However, in the rapid growing pediatric participants, our observations are still informative to provide clinical insights. Future work should focus on incorporating pathological participants who may have different patterns of physical activity and gait. Lastly, although our study was properly designed to ensure statistical power for validating the gait metrics, it still has a small sample size to be used as a reference dataset, particularly for specific age groups. Further efforts should focus on applying our methods to larger observational studies to build a more robust reference for gait and physical activity in pediatric populations.

## Conclusion

The use of wearable DHTs is acceptable to the pediatric population and can provide reliable measurement of gait for the pediatric population. DHT-measured gait and physical activity in free-living environments is well positioned to be used as an innovative and patient-centric approach to provide novel digital endpoints for pediatric clinical trials.

## Supplementary information

Supplementary Material

## Data Availability

Upon request, and subject to review, Pfizer will provide the data that support the findings of this study. Subject to certain criteria, conditions and exceptions, Pfizer may also provide access to the related individual de-identified participant data. See https://www.pfizer.com/science/clinical-trials/trial-data-and-results for more information.
