# Supplementary material for: Monitoring Activity and Gait in Children (MAGIC) using digital health technologies
Source: Pediatr Res. 2024 Mar 21;96(3):750–8. doi: 10.1038/s41390-024-03147-x (PMC11499282; doi:10.1038/s41390-024-03147-x)
Supplement: Supplementary file 1 — Supplementary Material [file 41390_2024_3147_MOESM1_ESM.pdf]

Supplementary Material for Monitoring Activity and Gait in Children (MAGIC) using Digital Health Technologies

Supplementary Table 1. Comfort and Wearability Questionnaires from In-Lab Portion

	Strongly Disagree	Disagree	Neutral	Agree	Strongly Agree
**The device is comfortable to wear.**					
ActiGraph Wrist	0	1 (2.5%)	5 (12.5%)	15 (37.5%)	19 (47.5%)
ActiGraph Lumbar	0	2 (5.0%)	4 (10%)	22 (55.0%)	12 (30%)
**The device is easy to put on.**					
ActiGraph Wrist	1 (2.5%)	2 (5.0%)	2 (5.0%)	13 (32.5%)	22 (55%)
ActiGraph Lumbar	0	1 (2.5%)	5 (12.5%)	16 (40%)	18 (45%)
**The device is easy to take off.**					
ActiGraph Wrist	0	1 (2.5%)	1 (2.5%)	16 (40%)	22 (55%)
ActiGraph Lumbar	0	0	2 (5.0%)	14 (35%)	24 (60%)
**The device is easy to wear.**					
ActiGraph Wrist	0	0	1 (2.5%)	19 (47.5%)	19 (47.5%)
ActiGraph Lumbar	0	1 (2.5%)	1 (2.5%)	19 (47.5%)	19 (47.5%)
**The device changes the way I move*.**					
ActiGraph Wrist	24 (60%)	13 (32.5%)	2 (5%)	0	1 (2.5%)
ActiGraph Lumbar	26 (65%)	13 (32.5%)	1 (2.5%)	0	0
**The device changes the way I behave*.**					
ActiGraph Wrist	29 (72.5%)	8 (20%)	2 (5%)	1 (2.5%)	0
ActiGraph Lumbar	30 (75%)	9 (22.5%)	1 (2.5%)	0	0
**I am willing to wear the device for 4 to 7 days.**					
ActiGraph Wrist	0	0	0	11 (27.5%)	29 (72.5%)
ActiGraph Lumbar	0	0	0	14 (35%)	26 (65%)
**I am willing to wear the device for more than 7 days.**					
ActiGraph Wrist	0	0	1 (2.5%)	14 (35%)	25 (62.5%)
ActiGraph Lumbar	0	0	2 (5%)	14 (35%)	24 (60%)
**If the device could follow my progress during a treatment, I would wear it.**					
ActiGraph Wrist	0	0	0	12 (30%)	28 (70%)
ActiGraph Lumbar	0	0	0	15 (37.5%)	25 (62.5%)
**If the device could follow my progress during a treatment, I would want my doctor to have access to my results.**					
ActiGraph Wrist	0	0	0	13 (32.5%)	27 (67.5%)
ActiGraph Lumbar	0	0	0	15 (37.5%)	25 (62.5%)

Supplementary Table 2. Comfort and Wearability Questionnaires from At-Home Portion

	Strongly Disagree	Disagree	Neutral	Agree	Strongly Agree
**The device is comfortable to wear.**					
ActiGraph Wrist	0	1 (2.5%)	1 (2.5%)	15 (37.5%)	23 (57.5%)
ActiGraph Lumbar	1 (2.5%)	4 (10%)	6 (15%)	18 (45.0%)	11 (27.5%)
**The device is easy to put on.**					
ActiGraph Wrist	0	2 (5.0%)	2 (5.0%)	15 (37.5%)	21 (52.5%)
ActiGraph Lumbar	0	2 (5.0%)	2 (5.0%)	13 (32.5%)	23 (57.5%)
**The device is easy to take off.**					
ActiGraph Wrist	0	1 (2.5%)	0	16 (40%)	23 (57.5%)
ActiGraph Lumbar	0	0	1 (2.5%)	12 (30%)	27 (67.5%)
**The device is easy to wear.**					
ActiGraph Wrist	0	0	1 (2.5%)	14 (35%)	25 (62.5%)
ActiGraph Lumbar	0	3 (7.5%)	8 (20%)	14 (35%)	15 (37.5%)
**The device changes the way I move*.**					
ActiGraph Wrist	32 (80%)	8 (20%)	0	0	0
ActiGraph Lumbar	22 (55%)	15 (37.5%)	0	3 (7.5%)	0
**The device changes the way I behave*.**					
ActiGraph Wrist	29 (72.5%)	10 (25%)	0	1 (2.5%)	0
ActiGraph Lumbar	26 (65%)	11 (27.5%)	1 (2.5%)	2 (5.0%)	0
**I am willing to wear the device for 4 to 7 days.**					
ActiGraph Wrist	0	0	0	13 (32.5%)	27 (67.5%)
ActiGraph Lumbar	1 (2.5%)	3 (7.5%)	0	18 (45%)	18 (45%)
**I am willing to wear the device for more than 7 days.**					
ActiGraph Wrist	0	1 (2.5%)	0	14 (35%)	25 (62.5%)
ActiGraph Lumbar	2 (5.0%)	3 (7.5%)	2 (5%)	17 (42.5%)	16 (40%)
**If the device could follow my progress during a treatment, I would wear it.**					
ActiGraph Wrist	0	0	0	12 (30%)	28 (70%)
ActiGraph Lumbar	0	1 (2.5%)	0	14 (35%)	25 (62.5%)
**If the device could follow my progress during a treatment, I would want my doctor to have access to my results.**					
ActiGraph Wrist	0	0	0	10 (25%)	30 (75%)
ActiGraph Lumbar	0	0	0	11 (27.5%)	29 (72.5%)

*Participant responses for ActiGraph wrist and lumbar devices will be compared to assess the participants' preference for device location. Due to the ordinal nature of the responses, a 0 to 4 scale will be assigned to each option (i.e. 0 = strongly disagree, and 4 = strongly agree), except for questions "The device changes the way I move" and "The device changes the way I behave", where the order of the scale should be reversed (i.e. 0 = strongly agree, and 4 = strongly disagree) to be consistent with the ranking of other questions.
